# Exploratory model−based optimizing isavuconazole dosing regimens for *Aspergillus* spp. in adult patients according to serum albumin levels: a pharmacokinetic/pharmacodynamic analysis using Monte Carlo simulation

**DOI:** 10.3389/fcimb.2026.1737154

**Published:** 2026-06-18

**Authors:** Xiao-Chen Wei, Ming-Feng Zhao, Hai-Rong Lyu, Xia Xiao

**Affiliations:** 1 Department of Pharmacy, Tianjin First Central Hospital, Tianjin, China; 2 Department of Hematology, Tianjin First Central Hospital, Tianjin, China

**Keywords:** *Aspergillus* spp., isavuconazole, Monte Carlo simulation, optimizing, pharmacokinetic/pharmacodynamic, serum albumin

## Abstract

**Objectives:**

Serum albumin strongly influences isavuconazole pharmacokinetics due to its high plasma protein binding. Hypoalbuminemia, common in critically ill and immunocompromised patients, may cause subtherapeutic concentrations with standard isavuconazole dosage. This study aimed to optimize isavuconazole dosing regimens against *Aspergillus* spp. in adult patients based on serum albumin levels.

**Methods:**

A published population pharmacokinetic model of isavuconazole was used to simulate four dosing regimens stratified by serum albumin levels. Monte Carlo simulations calculated probability of target attainment (PTA) and cumulative fraction of response (CFR) based on the area under the concentration-time curve/minimum inhibitory concentration (AUC/MIC) ratios, with separate targets for immunocompetent and immunocompromised patients. PTA or CFR > 90% was considered optimal for a dosing regimen.

**Results:**

Based on PTA analysis for *Aspergillus* spp. with an MIC of 1 μg/mL, PTAs were 0%, 71.23%, 100%, and 100% in immunocompetent patients with mild hypoalbuminemia and 0%, 0.32%, 100%, and 100% in those with marked hypoalbuminemia for 100 mg, 200 mg, 300 mg, and 400 mg q24h, respectively, whereas in immunocompromised patients PTAs were 0%, 0%, 33.04%, and 100% with mild hypoalbuminemia and 0%, 0%, 0%, and 100% with marked hypoalbuminemia at the same doses, supporting 300 mg q24h for immunocompetent hypoalbuminemic adults and 400 mg q24h for immunocompromised counterparts. Furthermore, the CFR analysis demonstrated that the 200 mg q24h regimen was adequate against *A. terreus* and *A. versicolor*. Additionally, the 300 mg q24h regimen was recommended for immunocompetent adult patients with hypoalbuminemia in the treatment of *A. fumigatus* and *A. flavus*; conversely, immunocompromised adult patients with hypoalbuminemia necessitated the 400 mg q24h dosage for these pathogens. However, the majority of simulating dosing regimens failed to achieve the desired CFR values against *A. niger*.

**Conclusions:**

These PK/PD simulations support isavuconazole dose optimization for hypoalbuminemic adults with aspergillosis and require prospective clinical validation.

## 1 Introduction

Invasive aspergillosis (IA), caused predominantly by *Aspergillus* species, represents a significant opportunistic fungal infection with high morbidity and mortality, especially among immunocompromised populations such as patients with hematologic malignancies and those undergoing hematopoietic stem cell transplantation (HSCT) or solid organ transplantation (Iyadorai et al., 2024; Lien et al., 2021; Fernández-Ruiz, 2024). These patients are particularly vulnerable due to their compromised immune defenses, which facilitate fungal colonization and invasion, leading to severe and often fatal infections. The global incidence of invasive fungal diseases, including IA, has increased substantially in recent decades, paralleling advances in cancer therapies and transplantation procedures that, while life-saving, induce profound and prolonged immunosuppression (Thompson et al., 2024). Among these, IA is recognized as a major cause of infectious complications in hematologic malignancy and transplant recipients, with reported incidences ranging widely but consistently highlighting a substantial burden of disease (Walsh et al., 2025).

Isavuconazole, a second-generation triazole antifungal agent, has emerged as a pivotal therapeutic option in the management of invasive fungal infections (IFIs), which have shown a marked increase in incidence over recent decades, particularly among immunocompromised and critically ill patient populations (Thompson et al., 2024). As an advanced-generation triazole, isavuconazole exhibits broad-spectrum antifungal activity against yeasts, molds, and dimorphic fungi, including challenging pathogens such as *Aspergillus* species and mucorales, positioning it as a first-line treatment for invasive aspergillosis and mucormycosis (Ellsworth and Ostrosky-Zeichner, 2020). Its pharmacokinetic (PK) profile, characterized by high oral bioavailability, a prolonged half-life, and linear, predictable disposition, underpins its clinical utility and distinguishes it from earlier triazoles such as voriconazole and posaconazole (Candel et al., 2025; Lewis et al., 2022). The favorable safety profile, including reduced hepatotoxicity and neurotoxicity, and fewer drug-drug interactions further enhance its suitability for use in complex patient cohorts, including those with renal impairment, mild to moderate hepatic dysfunction, and pediatric populations (Candel et al., 2025; Segers et al., 2024).

Despite these advantages of isavuconazole, interindividual variability in pharmacokinetics has been documented, particularly in special populations such as critically ill patients, those undergoing extracorporeal membrane oxygenation (ECMO), or renal replacement therapies, where altered drug clearance and distribution volumes may necessitate therapeutic drug monitoring (TDM) and dose adjustments (Peña-Lorenzo et al., 2025a; Mertens et al., 2023; Doménech-Moral et al., 2025). A crucial aspect influencing isavuconazole pharmacokinetics is its high plasma protein binding, traditionally assumed to be approximately 99%, which affects the unbound, pharmacologically active fraction of the drug (Jansen et al., 2023). Low serum albumin levels, or hypoalbuminemia, are frequently encountered in patients with severe infections, critical illness, hepatic dysfunction, and other chronic conditions. Hypoalbuminemia can substantially impact the pharmacokinetics of highly protein-bound drugs like isavuconazole by decreasing protein binding, which may increase the free fraction of the drug, alter its volume of distribution, and modify clearance rates. In hematologic patients, for instance, serum albumin has been identified as significant covariates influencing isavuconazole clearance, highlighting the clinical relevance of protein status in dosing considerations (Peña-Lorenzo et al., 2025b). Moreover, in critically ill patients, hypoalbuminemia combined with other factors such as ECMO support and renal replacement therapy contributes to marked variability in isavuconazole exposure, often resulting in subtherapeutic concentrations that compromise antifungal efficacy (Peña-Lorenzo et al., 2025a; Mertens et al., 2023; Doménech-Moral et al., 2025; Bienvenu et al., 2025; Bolcato et al., 2022). These findings emphasize the necessity for individualized dosing strategies to optimize therapeutic outcomes based on the patient’s albumin level.

The pharmacokinetic/pharmacodynamic (PK/PD) index serves as the core basis for evaluating antifungal efficacy and guiding optimal dosing, and the ratio of the 24-hour area under the concentration-time curve to the minimum inhibitory concentration (AUC_0-24_/MIC) has been validated as the primary PK/PD index for isavuconazole against *Aspergillus* spp. In research involving immunocompetent hosts, Seyedmousavi et al. demonstrated through an immunocompetent murine model of disseminated aspergillosis that the AUC_0_;–_24_/MIC index could robustly predict survival outcomes in infected mice: the effective target was 50.5 based on the Clinical and Laboratory Standards Institute (CLSI) standards (Seyedmousavi et al., 2015). For immunocompromised host models, which better recapitulate clinical high-risk patient populations, Kovanda et al. further defined the PK/PD targets for immunocompromised states using a persistently neutropenic rabbit model of invasive pulmonary aspergillosis, in which an AUC_0-24_/MIC of 79.7 (per CLSI standards) was identified as the critical exposure threshold (Kovanda et al., 2016a). These well-defined PK/PD indices and targets provide a quantitative framework to assess exposure - efficacy relationships and support individualized dosing decisions for isavuconazole in clinical practice. However, existing PK investigations have largely overlooked the integration of pharmacodynamic (PD) principles, which may limit the accurate optimization of dosing regimens for patients with invasive aspergillosis. Therefore, the purpose of our study was to utilize a PK/PD model and Monte Carlo simulation (MCS) to calculate the probability of target attainment (PTA) and the cumulative fraction of response (CFR) in order to choose optimal dosing regimens of isavuconazole against *Aspergillus* spp. in adult patients based on different levels of albumin.

## 2 Methods

### 2.1 Pharmacokinetic parameters

PK data was obtained from a previously published population pharmacokinetic (PPK) analysis of isavuconazole in adult patients with hematologic disorders (Peña-Lorenzo et al., 2025b). The source study was a prospective, uncontrolled investigation in adults treated with isavuconazole for IFIs and monitored through a TDM program. A one-compartment model with first-order absorption and elimination provided an adequate fit for 121 measured isavuconazole concentrations collected from 52 participants. The PPK model used in our study to calculate plasma clearance is described by the following equation:


$$\frac{CL}{F}=3.83\times {\left(\frac{BSA}{1.8}\right)}^{1.8}\times \left[1-0.19\times \left(\frac{ALB-31}{10}\right)\right]$$

where CL = plasma clearance (L/h), *F* = absolute bioavailability, BSA = body surface area (m^2^), and ALB = serum albumin (g/L). Since isavuconazole exhibits excellent oral absorption with an absolute bioavailability (F) approaching 100% (Schmitt-Hoffmann et al., 2016), we set the F to 1. To simplify the calculation, BSA was fixed at 1.8 m^2^ (the median value of the PPK study population reported by Peña-Lorenzo et al.). Isavuconazole exhibits dose-proportional pharmacokinetics, with the total area under the concentration-time curve (AUC) rising in direct proportion to the administered dose (Ananda-Rajah and Kontoyiannis, 2015). Thus, the AUC corresponding to any given dosing regimen can be calculated using the standard formula (AUC = *F* × Dose/CL). Four levels of albuminemia were defined following prior research classifications (Akirov et al., 2017; Moramarco et al., 2020): marked hypoalbuminemia (< 25 g/L), mild hypoalbuminemia (25–35 g/L), normal albuminemia (35–45 g/L), and hyperalbuminemia (> 45 g/L). All the serum albumin values for each category of albuminemia were obtained from an observational retrospective study (Moramarco et al., 2020) carried out on the entire hospital population of the “Fondazione Policlinico Tor Vergata-PTV” in 2018 (**Table 1**).

**Table 1:** Demographic and serum albumin values used in Monte Carlo simulations.

Categories of albuminemia	No. of patients	Proportion of patients aged over 65 years (%)	Albumin (g/L) ^a^
Marked hypoalbuminemia (< 25 g/L)	909	67.44	21.8 ± 2.9
Mild hypoalbuminemia (25–35 g/L)	4045	67.10	30.7 ± 2.7
Normal albuminemia (35–45 g/L)	4199	47.88	38.8 ± 2.6
Hyperalbuminemia (> 45 g/L)	214	21.50	46.4 ± 1.3

^a^ Value expressed as mean ± standard deviation.

### 2.2 Minimum inhibition concentration distribution of *Aspergillus* spp.

We used two sources of MIC distribution data, both determined using the CLSI broth microdilution procedure. The first was derived from Rybak et al (Rybak et al., 2015), providing detailed MIC data for six *Aspergillus* species (*A. fumigatus*, *A. flavus*, *A. nidulans*, *A. niger*, *A. terreus*, and *A. versicolor*) for isavuconazole. The second was derived from Jean et al (Jean et al., 2022), providing detailed MIC data for five *Aspergillus* species (*A. fumigatus*, *A. flavus*, *A. nidulans*, *A. niger*, and *A. terreus*) based on data from the Antimicrobial Testing Leadership and Surveillance (ATLAS) Program. The proportion of isolates falling into each MIC category were calculated (**Table 2**) and these distributions were used for MCSs to estimate the CFR for different dosing regimens in each category of albuminemia.

**Table 2:** Minimum inhibitory concentration (MIC) distributions of isavuconazole for *Aspergillus* spp.

Species	Total no. of isolates	% of isolates susceptible at an MIC (μg/mL) of:									
		0.03	0.06	0.125	0.25	0.5	1	2	4	8	16
MIC data reported by Rybak et al (Rybak et al., 2015)											
*A. fumigatus*	926	0.00	0.65	3.35	16.09	55.51	17.39	5.29	0.54	1.19	0.00
*A. flavus*	454	0.00	0.00	0.44	6.39	55.73	32.38	4.63	0.44	0.00	0.00
*A. nidulans*	106	0.00	6.60	48.11	17.92	16.04	11.32	0.00	0.00	0.00	0.00
*A. niger*	218	0.00	0.46	1.83	5.05	23.85	34.40	28.90	4.13	1.38	0.00
*A. terreus*	390	0.00	1.03	8.46	43.85	42.31	4.10	0.26	0.00	0.00	0.00
*A. versicolor*	75	6.67	4.00	26.67	32.00	22.67	5.33	1.33	0.00	1.33	0.00
MIC data reported by Jean et al (Jean et al., 2022)											
*A. fumigatus*	660	0.00	0.00	0.45	7.42	63.94	20.91	3.94	1.97	0.76	0.61
*A. flavus*	96	0.00	0.00	1.04	13.54	52.08	32.29	1.04	0.00	0.00	0.00
*A. nidulans*	26	7.69	15.38	50.00	26.92	0.00	0.00	0.00	0.00	0.00	0.00
*A. niger*	107	0.00	0.00	0.00	0.00	8.41	42.06	27.10	17.76	2.80	1.87
*A. terreus*	40	0.00	2.50	17.50	25.00	50.00	2.50	2.50	0.00	0.00	0.00

### 2.3 Pharmacodynamic targets for *Aspergillus* spp.

The AUC/MIC ratio best linked drug exposure with the antifungal activity based upon previous PK/PD investigations with triazoles (Bellmann and Smuszkiewicz, 2017). *The* immunocompetent PD target of isavuconazole against *Aspergillus* spp. was a total drug AUC_0-24_/MIC value of 50.5 based on an immunocompetent murine model of disseminated aspergillosis (Seyedmousavi et al., 2015). The immunocompromised PD target of isavuconazole against *Aspergillus* spp. was a total drug AUC_0-24_/MIC values of 79.7 based on a persistently neutropenic rabbit model of invasive pulmonary aspergillosis (Kovanda et al., 2016a).

### 2.4 Monte Carlo simulation

A MCS was performed with 10000 subjects using Crystal Ball software (version 7.2.2; Decisioneering Inc.; http://www.crystalball.com). During simulations, the mean and standard deviation of ALB were used in a normal distribution. The PD data (MICs) followed a discrete distribution. The simulated isavuconazole dosing regimens were 100, 200, 300 and 400 mg q24h. Isavuconazole is available in both oral and intravenous formulations, which show high bioequivalence due to its excellent oral absorption with an absolute bioavailability approaching 100% (Schmitt-Hoffmann et al., 2016). Therefore, the MCS results obtained in our study were applicable to either oral or intravenous administration. The intent was to calculate the PTA, defined as the probability that at least a specific value of a PK/PD index is achieved at a certain MIC, as well as the CFR, defined as the expected population PTA for a specific drug dose and a specific population of microorganisms. CFR was calculated using weighted summation with the following formula (Wei et al., 2020):


$$CFR=\sum_{i=1}^{n}PTA\left(MIC_{i}\right)\cdot p\left(MIC_{i}\right)$$

A PTA or CFR expectation value of ≥ 90% was considered optimal for a dosing regimen against a population of organisms (Masterton et al., 2005).

## 3 Results

### 3.1 Probability of target attainment analysis

**Figure 1:**
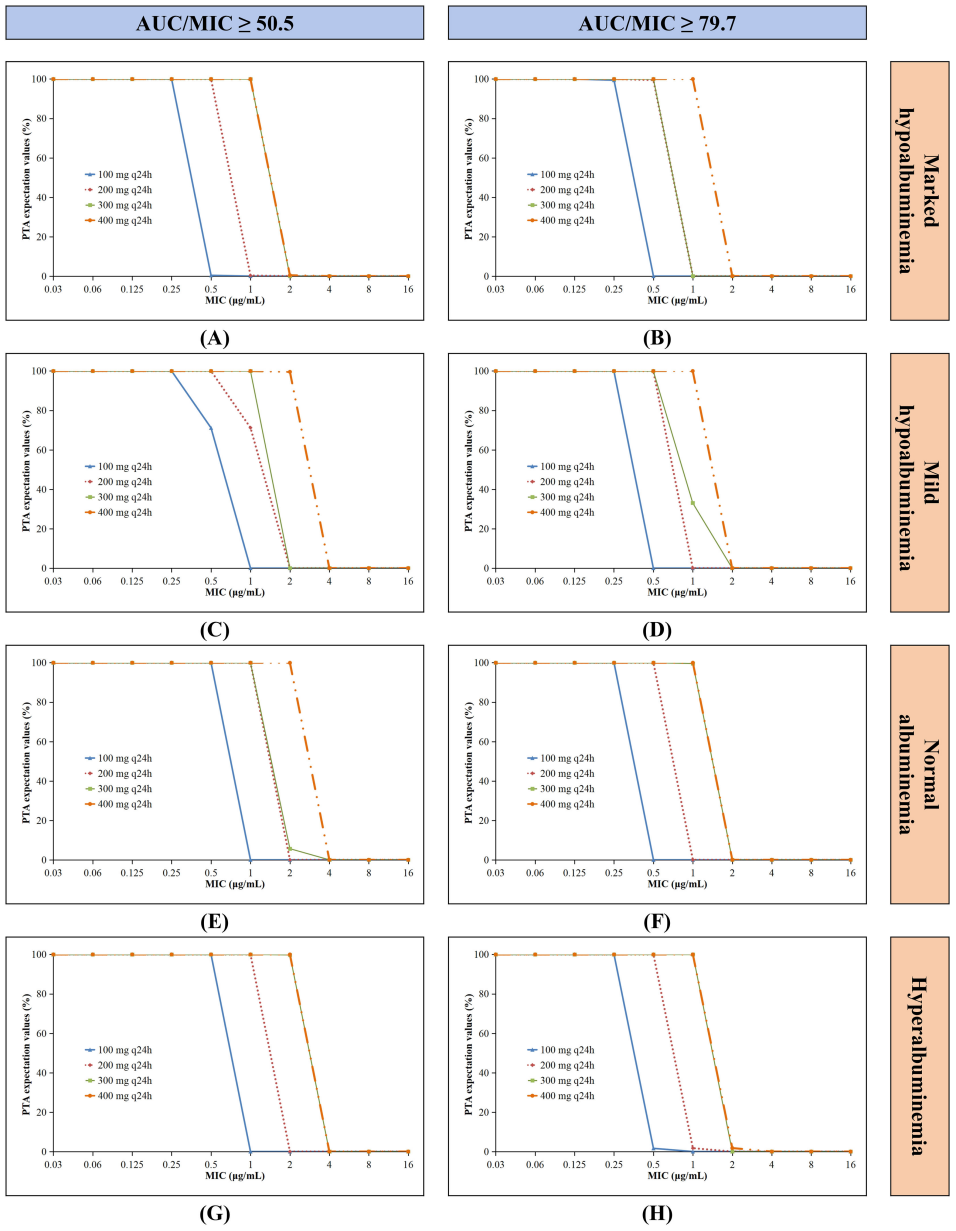
Probability of target attainment (PTA) of isavuconazole against *Aspergillus* spp. in adult patients with different albumin levels. MIC, minimum inhibitory concentration; AUC/MIC, total drug area under the concentration-time curve/minimum inhibitory concentration ratio. PTA values for the immunocompetent target of AUC/MIC ≥ 50.5 are presented for adult patients with marked hypoalbuminemia **(A)**, mild hypoalbuminemia **(C)**, normal albuminemia **(E)** and hyperalbuminemia **(G)**. PTA values corresponding to the immunocompromised target of AUC/MIC ≥ 79.7 are shown in adult patients with marked hypoalbuminemia **(B)**, mild hypoalbuminemia **(D)**, normal albuminemia **(F)** and hyperalbuminemia **(H)**. The PTA curves of the 300 mg and 400 mg q24h dosing regimens in **(A, F, G, H)** overlap. The PTA curves of the 200 mg and 300 mg q24h dosing regimens in **(B)** overlap.

**Figure 1** presents the probabilities of PK/PD target attainment by MIC for each isavuconazole dosing regimen against *Aspergillus* spp. in adult patients based on different albumin levels.

For patients with marked hypoalbuminemia, isavuconazole regimens of 100, 200, 300, and 400 mg q24h achieved PTAs ≥ 90% at MICs of ≤ 0.25, 0.5, 1, and 1 μg/mL, respectively, under the immunocompetent target (AUC_0-24_/MIC ≥ 50.5); corresponding MIC values for the immunocompromised target (AUC_0-24_/MIC ≥ 79.7) were 0.25, 0.5, 0.5, and 1 μg/mL (**Figures 1A, B**). In patients with mild hypoalbuminemia, 100, 200, 300 and 400 mg q24h of isavuconazole achieved PTAs ≥ 90% at MICs up to 0.25, 0.5, 1, and 2 μg/mL, respectively, for the immunocompetent target, and 0.25, 0.5, 0.5, and 1 μg/mL for the immunocompromised target (**Figures 1C, D**). Among patients with normal albumin levels, the maximum MIC values for PTAs ≥ 90% with 100, 200, 300, and 400 mg q24h were 0.5, 1, 1, and 2 μg/mL, respectively, for the immunocompetent target, and 0.25, 0.5, 1, and 1 μg/mL for the immunocompromised target (**Figures 1E, F**). For those with hyperalbuminemia, isavuconazole regimens of 100, 200–300 and 400 mg q24h provided PTAs ≥ 90% at MICs of ≤ 0.5, 1, 2, and 2 μg/mL, respectively, based on the immunocompetent target, and 0.25, 0.5, 1, and 1 μg/mL based on the immunocompromised target (**Figures 1G, H**).

### 3.2 Cumulative fraction of response analysis

**Table 3** presents the assessment of CFR expectation values for each isavuconazole dosing regimen against *Asper*gillus spp. in adult patients with different albumin levels based on MIC data reported by Rybak et al (Rybak et al., 2015).

**Table 3:** Cumulative fraction of response (CFR) expectation values (%) against various *Aspergillus* spp. for each isavuconazole dosage regimens in adult patients with different albumin levels based on MIC data reported by Rybak et al (Rybak et al., 2015).

Dosage regimens (mg qd)	CFR (%)											
	Immunocompetent target						Immunocompromised target					
	*A. fumigatus*	*A. flavus*	*A. nidulans*	*A. niger*	*A. terreus*	*A. versicolor*	*A. fumigatus*	*A. flavus*	*A. nidulans*	*A. niger*	*A. terreus*	*A. versicolor*
Marked hypoalbuminemia												
100	20.31	7.05	72.71	7.43	53.50	69.42	19.99	6.79	72.53	7.31	53.07	69.14
200	75.65	62.66	88.72	31.30	95.65	92.02	75.31	62.27	88.60	31.07	95.42	91.88
300	92.98	94.93	100.00	65.60	99.74	97.33	75.59	62.56	88.68	31.19	95.64	92.00
400	93.00	94.95	100.00	65.72	99.74	97.34	92.98	94.93	100.00	65.60	99.74	97.33
Mild hypoalbuminemia												
100	59.57	46.47	84.05	24.31	83.43	85.46	20.09	6.83	72.64	7.34	53.33	69.33
200	87.98	85.62	96.74	55.70	98.56	95.80	75.59	62.56	88.68	31.19	95.64	92.00
300	92.98	94.93	100.00	65.60	99.74	97.33	81.34	73.25	92.42	42.56	97.00	93.76
400	98.25	99.54	100.00	94.37	100.00	98.66	92.98	94.93	100.00	65.60	99.74	97.33
Normal albuminemia												
100	75.58	62.54	88.67	31.19	95.63	92.00	20.09	6.83	72.64	7.34	53.33	69.33
200	92.98	94.93	99.99	65.59	99.74	97.33	75.60	62.56	88.68	31.20	95.64	92.00
300	93.28	95.20	100.00	67.23	99.76	97.41	92.91	94.80	99.95	65.45	99.73	97.31
400	98.27	99.56	100.00	94.48	100.00	98.67	92.98	94.93	100.00	65.60	99.74	97.33
Hyperalbuminemia												
100	75.59	62.56	88.68	31.19	95.64	92.00	20.99	7.73	72.90	7.73	54.02	69.70
200	92.98	94.93	100.00	65.60	99.74	97.33	75.89	63.12	88.88	31.79	95.71	92.09
300	98.26	99.55	100.00	94.42	100.00	98.66	92.98	94.93	100.00	65.60	99.74	97.33
400	98.27	99.56	100.00	94.50	100.00	98.67	93.07	95.02	100.00	66.11	99.75	97.36

Grey, CFR values ≥ 90%.

For both immunocompetent and immunocompromised targets, the isavuconazole regimen of 200 mg q24h achieved a CFR ≥ 90% against *A. terreus* and *A. versicolor* in adult patients across all albumin level categories. In adult patients with normal albumin levels or hyperalbuminemia, the 200 mg q24h dosing demonstrated an optimal probability of antifungal success against *A. fumigatus* and *A. flavus* when targeting immunocompetent hosts. In contrast, a higher dose of 300 mg q24h was required to achieve a CFR ≥ 90% based on the immunocompromised target. Similarly, for patients with marked or mild hypoalbuminemia, a dosing of 300 mg q24h provided a CFR ≥ 90% against *A. fumigatus* and *A. flavus* under the immunocompetent target, while a larger dose of 400 mg q24h was necessary to meet the same threshold for the immunocompromised target. Regarding *A. nidulans*, among patients with mild hypoalbuminemia, normal albuminemia, or hyperalbuminemia, isavuconazole dosings of 200 mg and 300 mg q24h achieved target CFR values for immunocompetent and immunocompromised targets, respectively. For those with marked hypoalbuminemia, higher doses of 300 mg and 400 mg q24h were recommended for the immunocompetent and immunocompromised targets, respectively. In addition, doses of 300 mg and 400 mg q24h were sufficient against *A. niger* under the immunocompetent target in patients with hyperalbuminemia and those with mild hypoalbuminemia/normal albuminemia, respectively. However, none of the simulated isavuconazole regimens reached a CFR ≥ 90% against *A. niger* in patients with marked hypoalbuminemia (immunocompetent target) or in patients of any albumin category (immunocompromised target).

**Table 4** shows the CFRs of isavuconazole against various *Aspergillus* species across different albumin levels using MIC data from Jean et al (Jean et al., 2022). Overall, the CFRs trends were largely consistent with those observed in **Table 3**, with only minor discrepancies noted, primarily attributable to the distinct MIC distribution of *A. nidulans* and the absence of *A. versicolor* in this analysis.

**Table 4:** Cumulative fraction of response (CFR) expectation values (%) against various *Aspergillus* spp. for each isavuconazole dosage regimens in adult patients with different albumin levels based on MIC data reported by Jean et al (Jean et al., 2022).

Dosage regimens (mg qd)	CFR (%)									
	Immunocompetent target					Immunocompromised target				
	*A. fumigatus*	*A. flavus*	*A. nidulans*	*A. niger*	*A. terreus*	*A. fumigatus*	*A. flavus*	*A. nidulans*	*A. niger*	*A. terreus*
Marked hypoalbuminemia										
100	8.13	14.79	100.00	0.03	45.20	7.83	14.50	99.84	0.00	44.85
200	71.89	66.77	100.00	8.55	95.01	71.49	66.40	100.00	8.37	94.74
300	92.73	98.96	100.00	50.47	97.50	71.82	66.67	100.00	8.41	95.00
400	92.74	98.96	100.00	50.59	97.51	92.73	98.96	100.00	50.47	97.50
Mild hypoalbuminemia										
100	53.36	51.63	100.00	5.98	80.57	7.88	14.58	100.00	0.00	45.00
200	86.71	89.67	100.00	38.37	96.78	71.82	66.67	100.00	8.41	95.00
300	92.73	98.96	100.00	50.47	97.50	78.73	77.34	100.00	22.31	95.83
400	96.65	100.00	100.00	77.45	99.99	92.73	98.96	100.00	50.47	97.50
Normal albuminemia										
100	71.81	66.66	100.00	8.41	94.99	7.88	14.58	100.00	0.00	45.00
200	92.72	98.95	100.00	50.46	97.50	71.82	66.67	100.00	8.42	95.00
300	92.95	99.02	100.00	52.00	97.64	92.64	98.82	100.00	50.29	97.49
400	96.67	100.00	100.00	77.56	100.00	92.73	98.96	100.00	50.47	97.50
Hyperalbuminemia										
100	71.82	66.67	100.00	8.41	95.00	8.91	15.43	100.00	0.14	45.81
200	92.73	98.96	100.00	50.47	97.50	72.18	67.23	100.00	9.14	95.04
300	96.66	100.00	100.00	77.50	99.99	92.73	98.96	100.00	50.47	97.50
400	96.67	100.00	100.00	77.57	100.00	92.80	98.98	100.00	50.95	97.54

Grey, CFR values ≥ 90%.

## 4 Discussion

This study represents an exploratory, model−based PK/PD investigation using MCS to optimize isavuconazole dosing regimens against *Aspergillus* spp. in adult patients stratified by serum albumin levels. To our knowledge, this is the first study to employ PK/PD−based MCS for individualized isavuconazole dose optimization according to serum albumin levels in this population. To underpin this optimization, we selected a well-suited PPK model (Peña-Lorenzo et al., 2025b), which was developed based on 52 adult hematological patients with IFIs who were receiving isavuconazole, and a considerable proportion of these patients presented with hypoalbuminemia. Importantly, the model validation results from the original study, which incorporated assessments using goodness-of-fit plots, prediction-corrected visual predictive check (pcVPC) and bootstrap procedures, confirmed the model’s robust predictive performance and thus provide a solid, reliable foundation for the MCS in our present study. This model comprehensively integrates key covariates affecting isavuconazole PK, especially serum albumin as our core covariate, and it rigorously verifies that serum albumin is a critical factor influencing the clearance of isavuconazole. Its core advantage is its capacity to quantify the relationship between serum albumin and isavuconazole PK, which is highly consistent with our primary objective of optimizing isavuconazole dosing regimens according to serum albumin levels. In contrast, other existing isavuconazole PPK models were either established using healthy volunteers (Desai et al., 2016, Desai et al., 2019; Shirae et al., 2023) or did not include serum albumin as a covariate (Kovandaetal.,2016b; Martín-Cerezuela et al., 2024), making our selected model the most suitable for the present study.

Our PTA analysis results demonstrated that the clinically recommended dosing regimen (200 mg q24h) was appropriate for immunocompetent adult patients with normal albuminemia or hyperalbuminemia when treating *Aspergillus* spp. isolates with MICs ≤ 1 μg/mL — consistent with the susceptibility breakpoint established by EUCAST (European Committee on Antimicrobial Susceptibility Testing) and the FDA (U.S. Food and Drug Administration) for *A. fumigatus*. However, this regimen was insufficient for adult patients with mild hypoalbuminemia (PTA: 71.23%) and markedly insufficient for those with marked hypoalbuminemia (PTA: 0.32%). Emerging evidence indicates that isavuconazole pharmacokinetics can be significantly influenced by host pathophysiological conditions, including critical illness, hematologic disorders, and organ dysfunction (Peña-Lorenzo et al., 2025a, Peña-Lorenzo et al., 2025b; Li et al., 2025). In particular, serum albumin levels have been identified as key covariates affecting isavuconazole clearance, with hypoalbuminemia associated with increased drug clearance and reduced plasma concentrations (Peña-Lorenzo et al., 2025b). This is clinically relevant as low serum albumin, or hypoalbuminemia, is a common pathological state in critically ill patients, those with liver or renal impairment, and in malnourished individuals, all of whom represent a substantial proportion of patients receiving antifungal therapy (Peña-Lorenzo et al., 2025a; Li et al., 2025). Hypoproteinemia can alter the pharmacokinetics of highly protein-bound drugs like isavuconazole by reducing plasma protein binding, thereby increasing the free (active) drug fraction, potentially enhancing clearance and modifying tissue distribution (Peña-Lorenzo et al., 2025b). These changes may lead to subtherapeutic drug exposure, complicating dosing strategies and clinical outcomes. A recent retrospective multicenter study by Bienvenu et al. (2025), involving 102 patients admitted to hematology units or intensive care units (ICU), identified albumin levels on the day of TDM as a key factor influencing isavuconazole exposure. Underexposure to isavuconazole was frequent in these population, especially in ICU patients with hypoalbuminemia. Consequently, the conventional standard dose of isavuconazole (200 mg q24h) is inadequate in the treatment of *Aspergillus* spp. for patients with hypoalbuminemia, which is consistent with our PK/PD results. Moreover, isavuconazole exhibits a linear and proportional PK profile (Lewis et al., 2022), prompting us to simulate high-dose regimens in the present study. At an MIC of 1 μg/mL, our simulation results revealed that the PTA increased from 71.23% to 100% in patients with mild hypoalbuminemia and from 0.32% to 100% in those with marked hypoalbuminemia when the isavuconazole dosing was adjusted from 200 mg to 300 mg q24h. Therefore, our simulations suggest that a once-daily dose of 300 mg isavuconazole may be a potential strategy to improve target attainment in immunocompetent adult patients with hypoalbuminemia; this hypothesis should be tested in prospective clinical studies.

Neutropenia and immunosuppression represent pivotal risk factors for the development of IFIs, particularly invasive mold infections caused by *Aspergillus* species. These fungal pathogens preferentially affect hosts with impaired innate and adaptive immunity, such as patients undergoing chemotherapy for hematologic malignancies, recipients of solid organ or HSCT, and individuals receiving prolonged corticosteroid or other immunosuppressive therapies (Thompson et al., 2024). In clinical settings, hypoalbuminemia is frequently observed in neutropenic and immunocompromised patients, reflecting malnutrition, systemic inflammation, or protein-losing states common in critical illness and malignancy (Bolcato et al., 2022). A prior *post-hoc* analysis of the Phase 3 SECURE trial, conducted by Kontoyiannis et al (Kontoyiannis et al., 2018), revealed that the 42-day all-cause mortality rate reached 45.0% among neutropenic patients with IA who were treated with the standard dose of isavuconazole (200 mg q24h). Therefore, isavuconazole dose optimization is essential for neutropenic patients with IA, particularly those complicated with hypoproteinemia, as it helps enhance antifungal efficacy and reduce mortality rates. Our results suggested that isavuconazole administered at 300 mg q24h was effective against *Aspergillus* spp. with an MIC of 1 μg/mL in immunocompromised adult patients with normal albuminemia or hyperalbuminemia, while a higher dose of 400 mg q24h was required for those with mild to marked hypoalbuminemia. Our simulation results further generate the hypothesis that immunocompromised patients with IA may require higher isavuconazole doses compared to immunocompetent patients with equivalent albumin levels, a prediction that requires clinical confirmation. This is due to the fact that the former necessitates a higher PK/PD target value to achieve optimal therapeutic efficacy.

Furthermore, we utilized MCSs to estimate the CFR of each isavuconazole regimen against various *Aspergillus* species, including *A. fumigatus*, *A. flavus*, *A. nidulans*, *A. niger*, *A. terreus*, and *A. versicolor*, using MIC data from the study reported by Rybak et al (Rybak et al., 2015). Our stimulating results revealed that currently approved isavuconazole regimen (200 mg q24h) exhibits consistent effectiveness against *A. terreus* and *A. versicolor* in adult patients, regardless of their immune status (immunocompetent vs. immunocompromised) or albumin levels. For *A. nidulans*, the isavuconazole doses of 300 mg q24h and 400 mg q24h were suitable for immunocompetent and immunocompromised adult patients with marked hypoalbuminemia, respectively. For patients with other albumin level profiles, a 200 mg q24h regimen was sufficient for immunocompetent adults, while 300 mg q24h was adequate for immunocompromised adults. In addition, in the treatment of infections caused by *A. fumigatus* or *A. flavu*s (two common species of the *Aspergillus* spp.), the standard isavuconazole regimen (200 mg q24h) is effective for immunocompetent adult patients with normal or elevated serum albumin levels. For patients with hypoalbuminemia (either mild or marked), a dosing adjustment to 300 mg q24h was recommended as the appropriate approach. In the management of immunocompromised patients infected with *A. fumigatus* or *A. flavu*s, the administration of isavuconazole should be individualized according to the patient’s serum albumin status, with the following dosing recommendations: 300 mg q24h for those with normal or hyperalbuminemia; and 400 mg q24h for those with hypoalbuminemia (either mild or marked). However, almost all the regimens were ineffective against *A. niger*, except for isavuconazole administered at a dose of 400 mg q24h in immunocompetent adult patients with mild hypoalbuminemia or normal albumin levels, and at a dose of 300 mg q24h in those with hyperalbuminemia. Therefore, our findings enable the provision of tailored isavuconazole dosing recommendations targeting the specific *Aspergillus* species implicated based on the patient’s immune status and albumin level. Although the MIC data reported by Rybak et al. (2015) were published approximately 10 years ago, long-term surveillance data from the SENTRY program (Pfaller et al., 2021) and the ATLAS program (Jean et al., 2022) demonstrated that the MIC_50_; and MIC_90_; values of the most prevalent *Aspergillus* species (*A. fumigatus* and *A. flavus*) against isavuconazole remained highly stable, with no obvious shifts in their distribution. To further validate the reliability and robustness of our findings, we additionally performed MCSs using the more recent MIC dataset from the ATLAS program (Jean et al., 2022), and the results were highly consistent with those based on data from Rybak et al (Rybak et al., 2015).

To date, no well-established or universally accepted toxic threshold for isavuconazole has been defined in large-scale clinical trials or clinical guidelines. A previous preliminary study (Furfaro et al., 2019) proposed tentative toxicity cut-off values (4.87-5.13 mg/L) based on a small single-center cohort of only 19 patients, among whom merely 6 (31.6%) experienced mild adverse events, predominantly gastrointestinal symptoms. Given the limited sample size and low incidence of adverse events, these thresholds are insufficiently validated and not widely recognized as authoritative. Moreover, clinical trials of isavuconazole have consistently demonstrated a favorable safety and tolerability profile. Commonly reported side effects included mild gastrointestinal symptoms and transient laboratory abnormalities, none of which led to serious adverse events or discontinuation of therapy (Sivasubramanian and Chandrasekar, 2022). Of note, two dose-escalation studies (Cornely et al., 2015; Shirae et al., 2022) have collectively confirmed the tolerability of isavuconazole at doses of up to 400 mg, with no serious adverse events reported. These findings validate the safety of high-dose isavuconazole regimens in adult patients. Notably, all simulated dosages in our study (up to 400 mg q24h) fall within the clinically approved and well-tolerated dosage range. Therefore, our dosing recommendations were rationally derived from PK/PD target attainment, and the optimized regimens are unlikely to reach toxic exposure levels in clinical practice. Further large-scale, prospective studies are warranted to establish a definitive and reliable toxic threshold for isavuconazole in the future.

There are several limitations to this study. First, although the selected PPK model showed good precision, robustness, and predictive performance as evidenced by goodness-of-fit plots, pcVPC, and bootstrap procedures, an external evaluation of the model could not be performed in selected PPK study due to the lack of additional patients. Second, given that BSA was initially fixed at 1.8 m² for the sake of calculation simplification, particular caution must be taken when interpreting the results for individuals with extremely small or extremely large BSA. Third, loading dosing regimens of isavuconazole were not simulated owing to the lack of PK parameters. Fourth, as the selected model was initially developed for hematological patients, the optimized dosing regimens derived from this study may have limitations in generalizability to other subpopulations (e.g., patients with hypoalbuminemia caused by non-hematological or non-critical illnesses), which will require further clinical validation. Fifth, the present analysis was based on total plasma concentrations of isavuconazole rather than unbound (free) concentrations. Given the high plasma protein binding (approximately 99%) of isavuconazole, the unbound fraction represents the pharmacologically active moiety. Although our model included serum albumin as a key covariate to indirectly reflect changes in protein binding and unbound drug exposure, validated PPK parameters for unbound isavuconazole in this population were not publicly available at the time of simulation. Future studies incorporating measurement of free isavuconazole exposure are warranted to further refine dosing recommendations in patients with hypoalbuminemia. Sixth, the local MIC distribution must be considered and must be updated periodically because of the emergence of regional drug resistance and the huge regional differences in MIC distribution. Finally, although our simulations provide PK/PD-informed dose adjustment strategies, large-sample prospective clinical studies incorporating simultaneous assessment of serum albumin levels and therapeutic drug monitoring of plasma isavuconazole concentrations are warranted to verify the clinical value of our findings, validate the efficacy of the proposed regimens, and support their implementation into routine clinical practice. Such a reverse translational approach would help clarify whether hypoalbuminemia induces clinically relevant alterations in isavuconazole exposure and PK/PD target attainment in real-world settings. Furthermore, correlating serum albumin concentrations, measured drug exposure, and clinical outcomes would further strengthen the translational significance and clinical applicability of our model-based dosing suggestions.

## 5 Conclusion

In conclusion, the currently approved isavuconazole dosing regimen is insufficient against *Aspergillus* spp. with an MIC of 1 μg/mL in adult patients with hypoalbuminemia. Our simulation-based findings suggest that immunocompetent patients with hypoalbuminemia may benefit from a 300 mg q24h regimen, whereas immunocompromised patients may require 300 mg q24h when albumin levels are normal or elevated, and 400 mg q24h in the setting of hypoalbuminemia. Furthermore, dosing strategies may need to be tailored to the specific *Aspergillus* species involved. Nevertheless, these PK/PD-based simulations are hypothesis-generating, and prospective clinical validation is required before translating these dosing recommendations into routine clinical practice. Therefore, further prospective clinical studies incorporating concurrent assessment of serum albumin and isavuconazole exposure are warranted to verify the conclusions drawn from these simulations.

## Data Availability

The raw data supporting the conclusions of this article will be made available by the authors, without undue reservation.
